# Diagnostic and prognostic molecular pathology of lymphoid malignancies

**DOI:** 10.1007/s00428-023-03644-0

**Published:** 2023-09-25

**Authors:** Falko Fend, Michiel van den Brand, Patricia JTA Groenen, Leticia Quintanilla-Martinez, Adam Bagg

**Affiliations:** 1 Institute of Pathology and Neuropathology and Comprehensive Cancer Center, University Hospital Tübingen, Tübingen, Germany; 2 Pathology-DNA, Location Rijnstate Hospital, Arnhem, the Netherlands; 3 Department of Pathology, Radboud University Medical Center, Nijmegen, the Netherlands; 4 Cluster of Excellence iFIT (EXC 2180) ‘Image Guided and Functionally Instructed Tumor Therapies’, Eberhard Karls University Tübingen, Tübingen, Germany; 5 Department of Pathology and Laboratory Medicine, Hospital of the University of Pennsylvania, Philadelphia, PA USA

**Keywords:** Lymphoma, Molecular diagnostics, Clonality, Mutational analysis, Next-generation sequencing

## Abstract

With the explosion in knowledge about the molecular landscape of lymphoid malignancies and the increasing availability of high throughput techniques, molecular diagnostics in hematopathology has moved from isolated marker studies to a more comprehensive approach, integrating results of multiple genes analyzed with a variety of techniques on the DNA and RNA level. Although diagnosis of lymphoma still relies on the careful integration of clinical, morphological, phenotypic, and, if necessary molecular features, and only few entities are defined strictly by genetic features, genetic profiling has contributed profoundly to our current understanding of lymphomas and shaped the two current lymphoma classifications, the International Consensus Classification and the fifth edition of the WHO classification of lymphoid malignancies. In this review, the current state of the art of molecular diagnostics in lymphoproliferations is summarized, including clonality analysis, mutational studies, and gene expression profiling, with a focus on practical applications for diagnosis and prognostication. With consideration for differences in accessibility of high throughput techniques and cost limitations, we tried to distinguish between diagnostically relevant and in part disease-defining molecular features and optional, more extensive genetic profiling, which is usually restricted to clinical studies, patients with relapsed or refractory disease or specific therapeutic decisions. Although molecular diagnostics in lymphomas currently is primarily done for diagnosis and subclassification, prognostic stratification and predictive markers will gain importance in the near future.

## Introduction

Hematopathology has always been at the forefront in using molecular studies as tools for scientific progress and diagnostic advances. From the very beginning, molecular findings such as the identification of recurrent translocations or detection of clonality have shaped both our understanding of lymphoma and their classification [39].

With the relatively recent introduction of massively parallel sequencing, there has been an explosion in the knowledge about the genetic landscape of lymphoid proliferations [20]. This also had an important impact on the two new classifications for lymphoma, the International Consensus Classification (ICC) and the WHO HAEM5 classification [2, 12], as well as on the diagnostic practice. Molecular studies have four main applications in lymphoma pathology: (1) *diagnostic*, for confirmation of a neoplastic lymphoproliferation and for disease subclassification; (2) *prognostic*, for patient stratification and as aid in therapy decisions; (3) *predictive*, for the identification of targets of therapy; and (4) for the detection of *measurable (formerly minimal) residual disease* (MRD). Genetic alterations including chromosomal translocations, copy number variations and mutations, gene expression profiles, and potentially epigenetic alterations can be used for diagnostic purposes. In addition, immunoglobulin (IG) and T-cell receptor (TR) gene rearrangements provide unique targets for detecting clonality in a lymphoproliferation. In this review, we summarize current molecular diagnostics in lymphoma, with a focus on clinical relevance. Acute lymphoblastic leukemia/lymphoblastic lymphoma, MRD detection, and detailed methodological aspects are beyond the scope of this review.

## Clonality analysis

Clonality analysis is an important technique in the diagnosis of lymphoid proliferations, which allows distinction between the immunogenetic variability in reactive lymphoid proliferations and the lack thereof in lymphoid neoplasia. The rationale of clonality analysis is based on the highly diverse spectrum of B and T cells which is generated to allow an adaptive immune response to the diverse array of antigens the individual may encounter. This diversity is generated by rearrangement and the junctional diversity of IG and TR genes. In a lymphoid proliferation, this diversity can be studied by multiplex PCR amplification of IG and TR genes. For conventional clonality analysis, this is followed by fragment size analysis of the resulting PCR products [46, 109]. In lymphoma, the neoplastic cells all originate from the same transformed cell and therefore have the same IG or TR rearrangement. Accordingly, a large proportion of the PCR products in a lymphoma sample will have the same size resulting in one or two clonal peaks in fragment size analysis. This contrasts with reactive lymphoid tissues, which consist of a diverse population of cells with a large diversity in IG and TR genes, generating a polyclonal Gaussian curve. To achieve a high sensitivity for the detection of clonality, multiple targets are tested (i.e., IGH and IGK; TRG and TRB) which provide complementary information.

In recent years, it has become possible to analyze the IG and TR products with next-generation sequencing (NGS), allowing a much more detailed analysis of the IG and TR rearrangements present in a sample [10, 90]. Within the EuroClonality-NGS working group, an assay was designed with novel primers that generate small amplicons, making it more suitable for formalin-fixed paraffin-embedded (FFPE) tissue [90]. The IG clonality analysis makes use of the complementarity of the different targets. PCR reactions are performed in three tubes (IGH VJ; IGH DJ; IGK VJ and IGKV/Intron-Kde), followed by library preparation for sequencing.

**Fig. 1:**
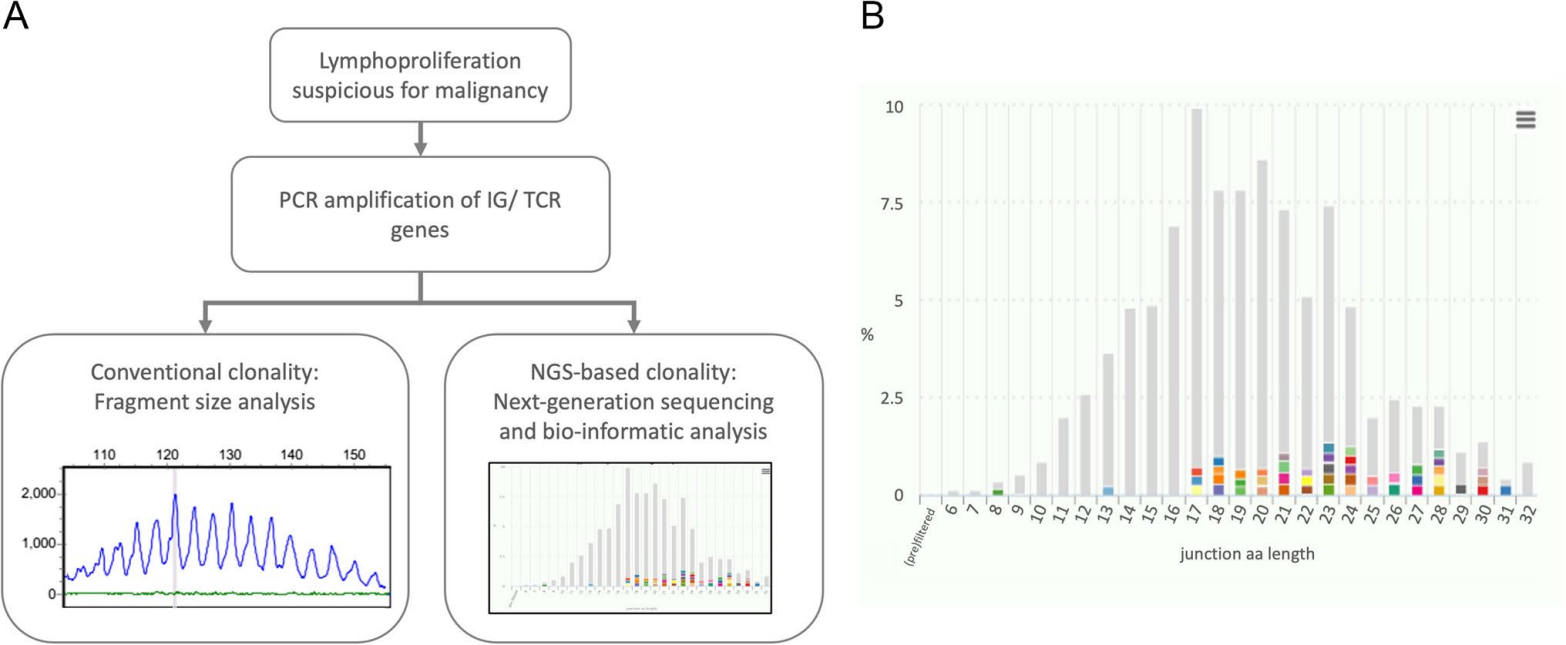
**A** Overview of conventional and NGS-based clonality analysis. From DNA isolated from a lymphoproliferation suspicious for neoplasia, IG and/or TR genes are amplified in a PCR reaction. For conventional clonality analysis, the PCR product is analyzed by fragment size analysis. For NGS-based clonality analysis, the PCR product is sequenced with a next-generation sequencing platform after which bio-informatic analysis is performed. **B** Example of an NGS-based clonality analysis result for IGH DJ with a polyclonal pattern, output from ARResT/Interrogate. The amino acid length of the junction is indicated on the *x-*axis, and the percentage of reads is indicated on the *y*-axis. The 50 most prominent clonotypes are indicated with different colors. Less frequent clonotypes in the background are shown in gray

To analyze the sequencing data from NGS-based clonality analysis, a bio-informatic analysis pipeline is used to identify clonotypes in the raw sequencing data [11, 18]. A clonotype is defined by the involved genes and the amino acid sequence of the junction between these genes. The distribution of the different clonotypes can then be viewed in tabular format or with different types of visualizations (Fig. 1).

Before interpreting, it is important to verify the quality of the results from NGS-based clonality analysis. DNA quality, DNA input, the lymphoid component, the number of cells suspicious for malignancy, the number of reads, and the number of clonotypes detected in the sample are important parameters. In particular, a pattern in which a limited number of clonotypes is present in a high percentage with a very limited background suggests a low template and should be signed out as non-evaluable. This low template pattern can be caused by poor DNA quality, few B-cells, or the presence of a prominent clone which cannot be amplified due to somatic hypermutation leading to primer mismatch.

**Fig. 2:**
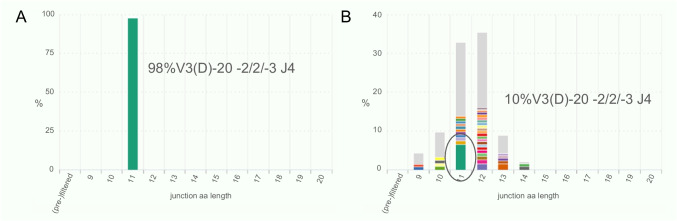
Assessment of clonal relationship between a mantle cell lymphoma in the lymph node (**A**) and the bone marrow (**B**). In these results from the IGK VJ target, a clear clone is detected in the lymph node in 98% of reads with a ratio between the #1 clonotype and the average of clonotypes #3–#7 of 363. The same clone is detected in the bone marrow in 10% of reads with a ratio of 1.8. Especially when looking for a known clonotype, NGS-based clonality analysis has a very high sensitivity

Similar to conventional clonality analysis, NGS-based clonality analysis can be used to distinguish reactive lymphoid proliferations from neoplasia. Studies with different assays have shown that NGS-based IG clonality analysis is at least as sensitive as conventional BIOMED-2 clonality analysis [6, 30, 71]. In conventional clonality analysis, small clones are often inapparent in the polyclonal background, especially if the size of the clone is near the center of the Gaussian curve. With NGS, small clones can be detected within the polyclonal background. In a technical feasibility study of the EuroClonality-amplicon-based NGS protocol, a lymphoma diluted in a polyclonal sample could still be detected as clonal with a limit of detection of 2.5%[90]. Sensitivity can be increased if the ratio between the dominant clonotype and the background, and replicate analyses are included in the assessment of the assay. If the purpose of the analysis is to search for the presence of an index clonotype, the sensitivity is even higher (Fig. 2).

This high sensitivity is very helpful in the study of lymphomas with few tumor cells in a prominent reactive background such as classic Hodgkin lymphoma (cHL) or T-cell/histiocyte-rich large B-cell lymphoma [106, 107]. Finally, the development of NGS-based clonality analysis has the potential to improve the diagnostic accuracy of notoriously difficult lymphoproliferations including skin lymphomas, early T-cell lymphomas, and lymphoproliferations in the context of immune deficiency and immune dysregulation. However, further studies are needed to determine the true potential and practical application of NGS-based clonality analysis in these diseases.

NGS-based clonality analysis has the potential to make interpretation of the results more objective as it provides quantitative and qualitative (i.e., actual sequence) data on the clonotypes that are present. Quantitative criteria for interpretation have been proposed which are based on a combination of the percentage of reads attributed to the most prominent clonotype and the ratio between the most prominent clonotype and the background [6, 30, 71]. When using a quantitative approach to NGS-based clonality interpretation, it is important to keep in mind that many variables are involved; results should always be interpreted in the histological and clinical context and taking into consideration results from other molecular analyses. Also, it must be emphasized that clonality does not equal malignancy; clonal results can be obtained in reactive conditions and lymphomas can generate a polyclonal result due to quality issues, few tumor cells, or an inability to detect the B-cell clone due to somatic hypermutation [47].

The sequences of the clonotypes from NGS-based clonality analysis allow for accurate clonal comparison. In patients with multiple synchronous or metachronous hematological malignancies, prognosis or treatment might be different depending on whether the lesions are clonally related or not [86]. Importantly, hematological malignancies that originate from the same clone may differ in morphology and immunophenotype which may even include a switch in lineage, sometimes due to so-called transdifferentiation [32]. In conventional clonality analysis, comparison based on clonal peak sizes can be inaccurate due to small sequence changes caused by mutations and technical artifacts [50, 70]. To conclude that two lymphoproliferations are clonally related generally requires identical peak sizes in at least two targets [34]. With NGS-based clonality analysis, the exact sequences can be compared which allows clonal comparison from a single target. In addition, NGS-based clonality analysis is very helpful to solve difficult cases with divergent clonal evolution. Concurrently or subsequently occurring lymphoid proliferations might have a shared ancestor clone as evidenced by shared IG/TR rearrangements for some targets, but they could at the same time have different IG/TR rearrangements on the other allele or in another target [7, 43]. Another application of NGS-based IG sequencing is the determination of the mutational status of the rearranged IG heavy chain gene (see below).

In conclusion, NGS-based clonality analysis opens up new possibilities for the diagnosis of lymphoid proliferations, but requires integration of results in the clinical, histological, and molecular context and requires knowledge about the potential and the technical and immunobiological pitfalls of IG/TR rearrangements. Recently, practical guidelines on the interpretation of NGS-based clonality results were described by the EuroClonality-NGS working group [108].

## Molecular studies in mature B-cell lymphomas

The differential diagnosis of small B-cell lymphomas is determined by the clinical presentation (leukemic, nodal, extranodal), morphology, and the immunophenotype. These features also guide the selection of molecular studies, including the detection of translocations and mutations, and their interpretation. Although many cases do not require these tests, mutational profiling is increasingly used for more precise subtyping and prognostic purposes (Table 1). Of note, even hallmark mutations such as *MYD88*^L265P^ or *BRAF*^V600E^ for lymphoplasmacytic lymphoma and hairy cell leukemia, respectively, are not completely specific, but rarely may be found in other small B-cell lymphomas.

**Table 1:** Genetic findings and biomarkers with potential relevance in small B-cell neoplasms

Disease entity	Genetic alterations	Diagnostic and clinical relevance
Chronic lymphocytic leukemia / small lymphocytic lymphoma	IGHV mutational status Del(11q), +12, del(13q), del(17p)	required, prognostic prognostic
	*TP53* mutation	required, before therapy initiation
	*NOTCH, SF3B1, BIRC, ATM, IGLV3-21* ^*R110*^, *FBXW7, XPO1*	prognostic
	*BTK, PLCG2, BCL2*	recommended for targeted therapy
Lymphoplasmacytic lymphoma	*MYD88* ^L265P/(L252P)^	Required, diagnostic
	*CXCR4* mutation	Required, predictive
Marginal zone lymphomas		
- Extranodal	*MALT1, BCL10, FOXP1* rearrangements, mutations in *TNAIP3* (ocular MZL) and *FAS* (cutaneous MZ-LPD)	Diagnostic, support diagnosis
- Splenic	del(7q) *KLF2, NOTCH2, TNFAIP3, TP53*	Diagnostic, in difficult cases
- Nodal	*KLF2, NOTCH2, PTPRD, BRAF, KMT2D*	Diagnostic, in difficult cases
Follicular lymphoma	t(14;18)(q32;q21); IGH::*BCL2*	Diagnostic
	*CREBBP, KMT2D, EZH2, MEF2B, RRAGC, TNFRSF14, EP300*	*EZH2* mutation predictive (Tazemetostat)
*- BCL2*-R neg, CD23+ FCL	*STAT6, CREBBP, SOCS1, TNFRSF14*	Diagnostic, in difficult cases
- Pediatric type FL	No t(14;18) *TNFRSF14, MAP2K1, IRF8*	Diagnostic, in difficult cases
Mantle cell lymphoma	t(11;14)(q13;q32); *CCND1::*IGH *TP53, ATM, BIRC3, KMT2D, NOTCH1/2*	cyclinD1 expression as surrogate *TP53* mutation poor prognosis
- cyclinD1- MCL	*CCND2-R, CCND3-R*	diagnostic by FISH or qRT-PCR
Hairy cell leukemia	*BRAF* ^V600E^	Diagnostic, for confirmation in difficult cases
HCL with IGHV4-34 and HCLv	*MAP 2K1* mutations (subset)	Diagnostic, separation from HCL
Multiple myeloma	*CCND* family translocations - t(11;14) *CCND1::*IGH - t(12;14) *CCND2::*IGH - t(8;14) *CCND3::*IGH *MAF* family translocations - t(14;16) IGH*::MAF* - t(8;14) *MAFA::*IGH - t(14;20) IGH*::MAFB* *NSD2 translocation* - t(4;14) *NSD2::*IGH Hyperdiploidy	Diagnostic, required for subtyping, prognosis and risk stratification
	1q gain, del(1p), *RAS*, *TP53* mutations, del(13), del(17p)	prognostic

*FCL*, follicle center lymphoma; *FL*, follicular lymphoma; *MCL*, mantle cell lymphoma; *HCL*, hairy cell leukemia; *HCLv*, hairy cell leukemia variant; *qRT-PCR*, quantitative reverse transcriptase PCR

### Chronic lymphocytic leukemia/small lymphocytic lymphoma (CLL/SLL)

Although the diagnosis of CLL/SLL is straightforward in most cases, genetic alterations are of great importance for prognostic stratification and influence therapy decisions. The presence or absence of somatic hypermutation in the rearranged IG heavy chain gene, taking a <98% identity with the germline sequence as cutoff, is of prognostic and therapeutic relevance [1]. In addition, some subsets of CLL with stereotyped B-cell receptors and CLL expressing IG lambda V3-21 with the R110 point mutation show distinct prognostic features [1, 66]. Although the cytogenetic and mutational landscape of CLL is highly diverse and shows differences between IGH-mutated and unmutated cases, alterations of *TP53*, usually bi-allelic (i.e., mutation and del17p) are the single most important prognostic and predictive parameter and need to be tested before initiation of therapy [64]. Of importance, even cases with small subclonal mutations (variant allele frequency (VAF) <10%) show a poorer outcome and response to therapy [85]. Mutational analysis of *BTK*, *PLCG2*, and *BCL2* is recommended for CLL under certain targeted therapies (ibrutinib and venetoclax) [64].

### Lymphoplasmacytic lymphoma (LPL)

LPL, in its classic form presenting as Waldenström’s macroglobulinemia, shows the canonical *MYD88*^L265P^ in 95 to 97% of cases, and *CXCR4* is mutated in 30 to 40% [91, 104]. IgM monoclonal gammopathy of undetermined significance (MGUS) and non-IgM LPL show these mutations at lower frequency. Especially for IgM MGUS and LPL with low tumor burden, sensitive techniques need to be employed for detection [22]. The presence or absence of these two mutations has a significant impact on clinical features and therapy response and should be determined in all LPL cases. Whereas the IgM-expressing multiple myeloma is universally negative, rare cases of splenic [74], nodal, and extranodal marginal cell lymphomas in the ocular adnexal region show *MYD88* mutations [76], as well as 2–3% of CLL, the latter cases associated with favorable biology [64].

### Marginal zone B-cell lymphomas

Due to the lack of a specific immunophenotype or a defining or characteristic genetic alteration, diagnosis of marginal zone lymphomas (MZL) relies on a combination of clinical, morphological, and phenotypic features, but mutational profiling and translocation detection may help to separate them from other small B cell lymphomas. A subset of MALT lymphomas shows recurrent translocations frequently involving the *MALT1* locus on 18q21; their frequency, as well as those of some recurrent mutations is strongly dependent on the primary organ manifestation [110]. In small B-cell lymphomas with nodal or bone marrow presentation, *KLF2*, *NOTCH1/2*, *PTPRD*, *BIRC*, *TRAF3*, *CARD11*, and *IRF8* mutations favor a diagnosis of MZL. Of note, nodal MZL rarely may show *MYD88* or *BRAF* mutations [49, 76, 96].

### Follicular lymphoma and variants

Conventional follicular lymphoma (FL) is characterized by the presence of a t(14;18)(q32;q21) translocation in about 85–90% of cases. The mutational profile is characterized by frequent alterations of epigenetic regulators, transcription factors, and others, whereas transformation is associated with a different set of genes, including *TP53* mutations and *MYC* translocations[44, 49]. Although risk stratification models incorporating mutations, such as the m7-FLIPI [75], have been proposed, they are strongly dependent on the type of therapy. Therefore, genetic profiling currently is not of clinical or diagnostic relevance in typical FL, except for the detection of *EZH2* mutations before treatment with the inhibitor tazemetostat [62]. However, it is useful for discerning FL variants, including the newly described *BCL2* rearrangement negative, CD23+ follicle center lymphoma [12, 67], which shows a high frequency of *STAT6* mutations, cutaneous follicle center lymphoma and pediatric type FL, which shows frequent alterations of *TNSFRSF14*, *MAP2K1*, and *IRF8*, distinct from adult FL [92].

### Mantle cell lymphoma

Mantle cell lymphoma (MCL) is characterized by the presence of the t(11;14) translocation, usually easily demonstrable by the surrogate marker cyclin D1 overexpression. MCL can be divided into the conventional subtype, derived from naïve B cells with unmutated IGHV genes, usually SOX11+, whereas the leukemic non-nodal type shows somatic hypermutated IGHV genes, lacks SOX11, and shows less genomic complexity [65]. Clinically relevant for both subtypes are *TP53* mutations, associated with a more aggressive clinical course, higher proliferation rate, and frequent blastoid morphology. The rare cyclin D1− MCL show rearrangements of *CCND2* or *CCND3*, which should be demonstrated by FISH or other techniques, since immunohistochemistry is unreliable in this setting [57].

### Other small B-cell lymphomas

Hairy cell leukemia is virtually universally characterized by the *BRAF*^*V600E*^ hotspot mutation, with the exception of cases using the IGHV4-34 family, which may show *MAP2K1* mutations and are similar to hairy cell leukemia variant [102].

### Plasma cell neoplasms

Multiple myeloma (MM) and its precursor lesion, non-IgM MGUS, show great genetic heterogeneity, with 45–50% showing trisomies of odd-numbered chromosomes, and 40–45% showing recurrent IGH translocations with a variety of partners, which are currently detected by interphase FISH [20]. Due to the prognostic impact of genetic alterations, the ICC has formally divided MM into cytogenetic subgroups [12]. Mutational profiling currently is not of diagnostic relevance outside of clinical studies, but an absence of *MYD88* mutations helps to exclude LPL [27].

### Diffuse large B-cell lymphoma and other large B-cell lymphomas

Diffuse large B-cell lymphoma (DLBCL) is both the most common and most diverse lymphoma occurring in adults, and a number of parameters, including but not restricted to clinical site and pathologic features, are used for subclassification. One of the earliest, and at the time most paradigm-shifting, approaches to applying molecular tools to the characterization of DLBCLs, particularly those that belong to the largest category of DLBCLs, not otherwise specified (NOS), occurred almost a quarter-century ago with the identification of two major forms based upon their putative cell-of-origin (COO) [4]. This original study employed gene expression profiling (GEP) using DNA microarrays to identify and divide most of these DLBCL, NOS cases into those of either germinal center B-cell (GCB) or activated B-cell (ABC) origin. This bifurcation also had prognostic connotations, in that the latter predicted for worse outcomes [113]. At a mechanistic level, GCB-DLBCLs tend to harbor mutations in chromatin-modifying genes while ABC-DLBCLs are more likely to display mutations that result in activation of the NFKB and BCR pathways [40, 115]. Notwithstanding the availability of a commercial RNA-based assay (Lymph2Cx) [116] that has yet to be widely adopted, translation of the COO strategy into routine clinical use has been difficult. This has led to the development of a number of surrogate immunohistochemical (IHC) algorithms that, unfortunately, do not perfectly reflect the GEP-based classification [35, 59]. While the binary use of COO, using IHC algorithms, has been retained in contemporary classification schemes and is used in diagnostic practice, it has become clear that this seemingly simple separation may not be sufficiently robust to capture the complexity of the molecular underpinnings of DLBCL to be of independent clinical and therapeutic value [19, 72].

**Fig. 3:**
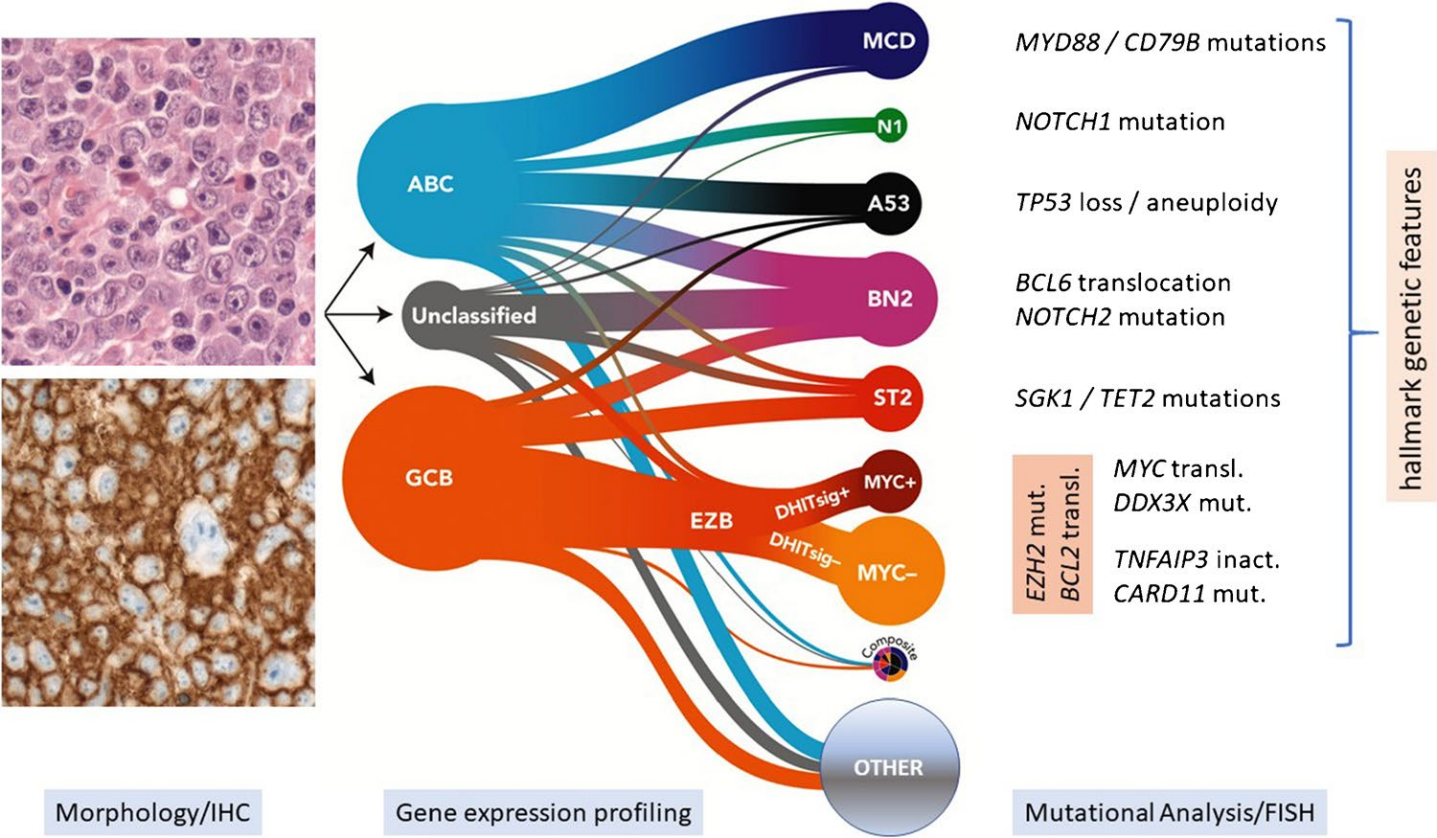
Molecular subtypes of DLBCL, NOS incorporating gene expression profiles and recurrent genetic alterations (modified according to [20]). The relationship between COO and the probabilistic assignments to genetics-based subgroups are shown. The size of the subgroup circles approximates the proportions of patients in each group. Tumors assigned with high confidence to ≥2 subgroups are assigned to the composite group, while ∼37% of tumors are not assigned to any subgroup with sufficient confidence (other). The hallmark genetic features are frequent within the respective subgroup but are not required for that assignment. Although capture of the total complexity would require a workup beyond current diagnostic standards, sequencing of a limited gene panel together with immunophenotyping and FISH for recurrent translocations can help to (tentatively) assign a significant subset of DLBCL, NOS to one of the molecular subgroups

This complexity has more recently been unraveled using a compendium of genomic approaches by different investigators that have independently largely converged into somewhat overlapping classifications identifying up to seven major subtypes of DLBCL [13, 45, 93] (Fig. 3). The complexity and comprehensive genetic testing required to unravel these different subtypes is currently not readily adoptable by routine diagnostic laboratories, but publicly available online tools such as LymphGen may be used to facilitate classification [87, 114]. Of note, the LymphGen tool does not subtype 30-40% of cases of DLBCL into any of the major groups, in contrast to other proposed molecular subclassifications of DLBCL which assign all evaluable cases to a specific category. Many of the key or defining features in the various clusters are already available for testing in some or even most routine settings, since they can be ascertained by a combination of FISH (for *BCL2* and *BCL6* translocations, and *TP53* loss) and targeted mutational analysis (evaluating *MYD88*, *CD79B*, *EZH2*, *CREBBP*, *KMT2D*, *NOTCH1*, *NOTCH2*, *TP53*, *SGK1*, *TET2*, and *SOCS1*, among others), although it is unlikely that such focused approaches fully capture the complexity of these subtypes. Nevertheless, a simplified 20-gene classifier has been described and has the potential to approximate the LymphGen approach to classify four main subgroups [60]. Conspicuously, and perhaps surprisingly, (almost entirely) absent as a major variable in the stratification of the different genomic categories of DLBCL is testing for a *MYC* translocation that has long been a key component of evaluation of this group of lymphomas. However, its presence does help refine one of the subgroups, and it remains an important initial genetic abnormality to evaluate in all aggressive B-cell lymphomas, particularly as a necessary first step in identifying the genetically-defined subtype of high-grade B-cell lymphoma (HGBL) of double-hit lymphoma (DHL), as discussed below. Provocatively, many of these major genomic subtypes of DLBCL share features evident in other, and often indolent, B-cell lymphomas such as marginal zone lymphoma, follicular lymphoma, and small lymphocytic lymphoma/chronic lymphocytic leukemia, as well as other defined DLBCLs, including those arising in extranodal immune-privileged sites or the mediastinum.

Another genetically defined LBCL entity is associated with an 11q aberration which in most cases harbors the combination of a proximal gain at 11q23.2-11q23.3 and distal loss at 11q24-qter, although some only show one of these features [88]. This lymphoma was previously classified as a Burkitt-like lymphoma but that designation has subsequently been revised since these cases, though morphologically and immunophenotypically sharing features with Burkitt lymphoma (BL), actually lack mutations that are typically evident in BL, as discussed below [31]. Commercially available 11q FISH probes are imperfect in identifying this defining aberration, and copy number array or other genomic analyses may be necessary to make this diagnosis. Notably, while classified as a LBCL by the ICC, WHO-HAEM5 has designated this as a form of HGBL [2, 12]. The rationale of the ICC for classifying the cases with 11q aberration as LBCL, rather than using the morphologically inspired term HGBL is based on their clinically less aggressive course and favorable outcome after therapy [31]. LBCLs with *IRF4* rearrangements are another subtype that requires genetic testing for its diagnosis, typically by FISH although they also harbor mutations of *IRF4* that may further support the diagnosis [80]. This entity is handled differently by ICC, which categorizes such cases within follicular lymphoma, given its overlapping features and typically indolent behavior, as compared with WHO-HAEM5, which groups this together with other LBCLs.

### High-grade B-cell lymphoma

One of the two originally designated entities of high-grade B-cell lymphoma (HGBL) was one with *MYC* and *BCL2* and/or *BCL6* translocations, which was also termed double-hit (DH) or triple-hit (TH) lymphoma. However, this has subsequently been split into two distinct categories (1) HGBL with *MYC* and *BCL2* rearrangements, which account for 80–90% of such cases; and (2) HGBL with *MYC* and *BCL6* rearrangements, which account for 10–20% of such cases, with substantial evidence allowing the former to be retained as a bona fide entity, while the latter seems to be more heterogeneous and has been relegated, by the ICC but not WHO, to a provisional entity [41]. Virtually all of the *MYC*+*BCL2* HGBLs are of GCB origin, whereas only around 50% of the *MYC*+*BCL6* HGBLs are. The mutational landscape of the two DHLs is also quite distinct. Since there are no pathologic features (including morphologic, COO, or proliferative index) that can reliably identify DHLs, all DLBCLs should be screened for these defining translocations. *MYC* break-apart probes (BAP) alone are inadequate for detecting all *MYC* translocations, and different commercially available probes vary in sensitivity [63]. The addition of dual-color dual fusion *MYC*::*IGH* FISH probes may increase the yield by detecting translocations missed by the *MYC* BAP [42]. While the *MYC* partner in Burkitt lymphoma (BL, see below) is essentially always an IG gene, this is not the case here, and non-IG genes are involved in many (at least 40%) cases, including *BCL6*, *IRF4*, and *PAX5* [16]. The suggestion that DHLs with *MYC* translocations involving IG have a worse prognosis appears to be unresolved [58, 84]. While DH *MYC*+*BCL2* HGBLs are defined by these translocations that are imperfectly identified by cytogenetics or FISH, they can also be identified, perhaps even more reliably, by transcriptional profiling and have variably been termed molecular high grade (MHG) or DHITSig wherein cryptic translocations, missed by cytogenetics or FISH, are evident, occurring in 20% of cases [24, 37, 94]. It remains to be determined when and whether such testing can be applied in the routine diagnostic setting. Such lymphomas that almost always are of GCB origin may also, together with Burkitt lymphoma (see below), fall under the umbrella term of dark-zone lymphomas or DZSig lymphomas, with these cases refining the DLBCL-COO classification by identifying patients within this category with an inferior prognosis [3]. HGBL that is not a DHL or THL, termed HGBL, NOS, while enriched in *MYC* translocations (seen in ~45%) do not have a consistent unifying genetic profile, and remains a problematic diagnosis that is heavily reliant on often subjectively determined morphologic features.

### Burkitt lymphoma

**Fig. 4:**
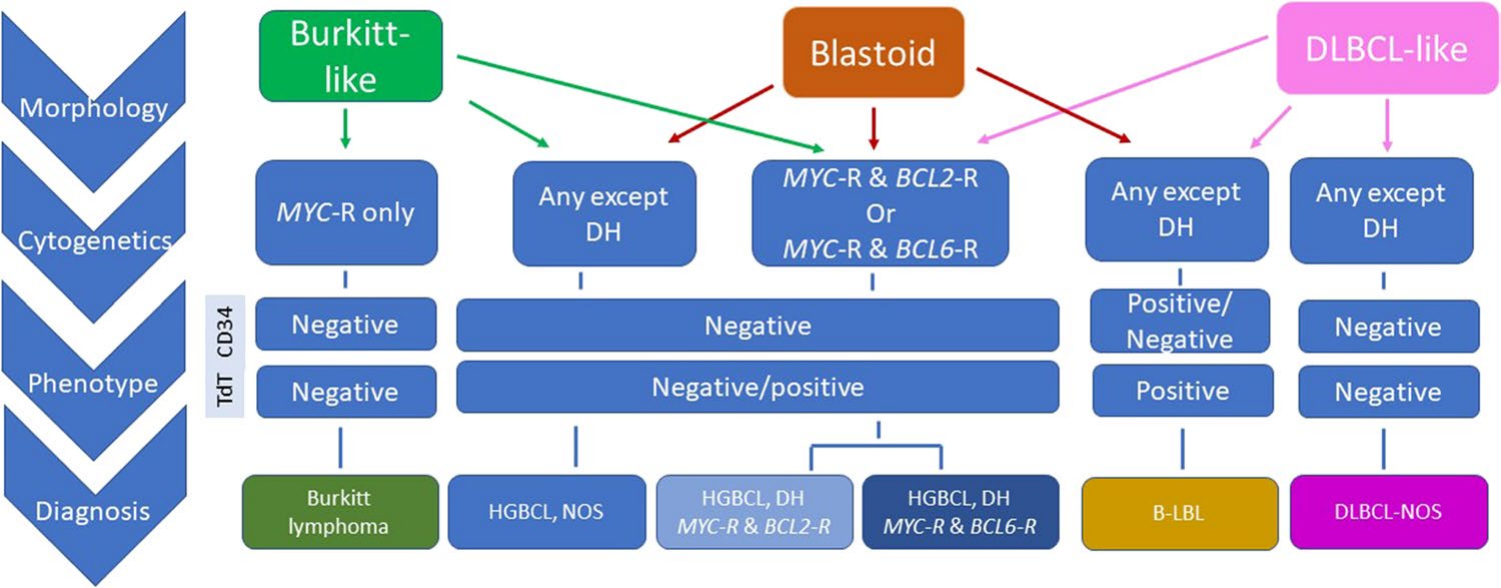
An approach to the use of FISH in aggressive B-cell lymphomas. This algorithm incorporating morphology, some immunohistochemical features, and detection of recurrent rearrangements allows subtyping of most aggressive B-cell lymphomas. Lymphomas with a Burkitt-like, blastoid, or large-cell morphology that have *MYC* with *BCL2* and/or *BCL6* rearrangements are categorized as HGBL, with either *MYC* and *BCL2* (with or without *BCL6* R) or *MYC* and *BCL6* R. HGBL, NOS lacks these double hits and may have either Burkitt-like or blastoid morphology. TdT expression, but not CD34, may be seen in HGBL. Burkitt lymphomas typically have a *MYC* R without *BCL2* or *BCL6* and a mature GC B-cell phenotype. B-lymphoblastic lymphomas are blastoid-appearing, usually TdT+ and frequently but not always CD34+. Cases with a large B-cell morphology that lack a DH, generally, are categorized as DLBCL, NOS, or as one of the more specific types of large B-cell lymphoma. The possibility of a large B-cell lymphomas with 11q aberrations should be considered particularly with a Burkitt-like morphologic appearance but with coarse apoptotic material in the tingible body macrophages and lack of a *MYC* R. Some cases, however, will more closely resemble a DLBCL. DLBCL, diffuse large B-cell lymphoma; R, rearrangement; HGBL: high-grade B-cell lymphoma; DH, double hit (modified according to [41])

The genetic hallmark of BL is a balanced translocation involving *MYC* and one of the three immunoglobulin genes (IGH, IGK, or IGL, in approximately 80%, 15%, and 5% of cases, respectively). The translocations occur in the germinal center in mature B-cells, either at the time of somatic hypermutation (SHM) or class switch recombination (CSR). In sporadic BL (see below), this event happens most often at the time of CSR (~75%), interestingly with a proclivity to involve IGHA, rarely seen in other *MYC*-translocated lymphomas [54]. While traditionally epidemiologically stratified into three types — sporadic, endemic, and immunodeficiency-associated BL — a perhaps more mechanistic (though imperfect) and somewhat overlapping approach might be to separate cases based upon the involvement (or not) of EBV. Thus, EBV-positive cases may have fewer, or at least different, additional driver mutations [33, 51]. BL lacking EBV tends to harbor canonical mutations affecting *ID3*, *TCF3*, and *CCND3* that are also more common in pediatric than in adult cases [82], while EBV-positive BL tends to be enriched in mutations affecting *DDX3X*, *GNA13*, and *FOX01* [101]. The mutations evident in EBV-negative cases are quite unique to BL, while different mutations in other GC-derived aggressive B-cell lymphomas such as those affecting genes encoding chromatin modifiers are not seen, which together might be used to bolster the diagnosis of BL in routine diagnosis [33]. BL that dichotomizes based upon EBV status and mutational profile also differ in their timing of the *MYC* translocation, occurring during SHM in EBV-positive BL and more typically during CSR in EBV-negative BL. Testing for *TP53* mutations is also of value in BL, providing important prognostic information for risk stratification [68]. Cases previously reported as TdT+ BL are no longer classified as BL and rather should be designated as B-lymphoblastic lymphoma/leukemia with *MYC* rearrangements. They differ from bona fide BL at three levels: (1) the *MYC::IGH* translocation occurs during VDJ recombination in the bone marrow; (2) they harbor *RAS* mutations, and lack those prevalent in BL; and (3) they do not express a functional B-cell receptor [111]. An approach to the initial FISH-based genetic workup of aggressive B-cell lymphomas, including DLBCL-NOS, HGBL, and BL is portrayed in Fig. 4, and current genetic tests are summarized in Table 2.

**Table 2:** Genetic findings and biomarkers with potential relevance in aggressive / large B-cell lymphomas

Disease entity	Genetic alterations and biomarkers	Diagnostic and clinical relevance
DLBCL, NOS *-* germinal center B-cell type (GCB) *-* activated B-cell type (ABC)	“Cell of origin” by immunohistochemistry or GEP, no “double hit” by FISH	Diagnostic, required for subtyping and separation from HGBL
	Molecular subtypes by mutation profiling	Panel sequencing, LymphGen algorithm [87, 114] or similar; prognostic and in part predictive
EBV+ DLBCL, NOS	EBV association	EBER1 ISH, diagnostic
High-grade BCL		
*-* with *MYC* and *BCL2-R*	- *MYC-R* and *BCL2-R* - *CREBBP, BCL2, KMT2D, EZH2* mutations	Diagnostic, required (FISH)
- with *MYC* and *BCL6-R*	*MYC-R* and *BCL6-R* - Heterogeneous mutations	Diagnostic, required (FISH)
- NOS	*MYC-R* in subset	Diagnosis of exclusion
Burkitt lymphoma	*MYC*-R with *IGH*, *IGK* or *IGL* genes	Diagnostic
	- *ID3, TCF3, DDX3X, CCND3, GNA13, TP53*	Optional, for difficult cases; *TP53* mutations prognostic
	- EBV association +/-	Desirable
Large/HG B-cell lymphoma with 11q aberration	11q23.2-q23.3 gain/11qter loss (including *ETS1* and *FLI1*)	Diagnostic, required (FISH, array CGH or similar)
	*GNA13, DDX3X, BTG2* mutations	
Large B-cell lymphoma with IRF4-R*	*IRF4*-R and/or *IRF4* mutations	Diagnostic, required; *IRF4* mutations in cases with cryptic translocation

*HGBL*, high-grade B-cell lymphoma; *ISH*, in situ hybridization; *FISH*, fluorescence in situ hybridization

*not considered an aggressive lymphoma by ICC

## Classic Hodgkin lymphoma

Although the genetic landscape of classic Hodgkin lymphoma (CHL) and also nodular lymphocyte-predominant Hodgkin/B-cell lymphoma has been elucidated in the recent years, mutational profiling currently plays no significant role in practical diagnosis [112]. Of note, detection of B-cell clonality can be achieved in a significant subset of cases with modern protocols, and therefore, clonality studies are not suited to differentiate between Hodgkin lymphoma and other B-cell lymphomas with CHL-like morphology. Conversely, the detection of T-cell clonality and mutations characteristic for TFH lymphoma (see below) may aid in differentiating T-cell lymphomas with Reed-Sternberg cells from CHL [103].

## Peripheral T-cell lymphomas

Peripheral T-cell lymphoma (PTCL) represents a heterogenous group of lymphoid malignancies, difficult to diagnose that constitutes approximately 10% of all lymphomas. PTCLs are divided according to their clinical presentation and site of involvement in leukemic, nodal, extranodal, and cutaneous [99]. The current ICC and WHO HAEM5 lymphoma classifications, recognize 33 and 36 diseases entities, respectively [2, 12]. The classification of PTCL in both lymphoma classifications is based on morphology and immunophenotype; however, this might change in the future since characteristic genetic alterations are increasingly recognized in PTCL. In contrast to B-cell lymphomas, clonality analysis is often needed to confirm a neoplastic T-cell proliferation with the caveat that finding a TR clonal rearrangement, *per se*, is not synonymous of a T-cell neoplasm.

### Genetic alterations with potential relevance for the diagnosis of nodal T-cell lymphomas

A predominant genetic alteration has been identified in only a minority of nodal T-cell lymphomas, such as *ALK* or *DUSP22* rearrangement (R) in anaplastic large T-cell lymphoma (ALCL). Although most nodal T-cell lymphomas have diverse molecular alterations, there are commonly affected cellular processes and signaling pathways [26]. In general, T-cell lymphomas are characterized by alterations in the TR and JAK/STAT signaling pathways, as well as mutations in epigenetic regulators [20]. These genetic alterations might have diagnostic, prognostic, or predictive impact in different entities (Table 3).

**Table 3:** Genetic findings and biomarkers with potential relevance in nodal T-cell lymphomas

Disease entity	Genetic alterations and biomarkers	Diagnostic and clinical relevance
ALCL, ALK positive	*ALK*-R (FISH)	Diagnostic, not required, prognosis: better than ALCL ALK-negative
	ALK (IHC) CD30 (IHC)	Required for diagnosis, therapeutic target Required for diagnosis, therapeutic target
	*NOTCH1* mutations	Potential therapeutic target
ALCL, ALK negative	CD30 (IHC)	Diagnostic, required
	*DUSP22*-R (FISH)	Diagnostic, recommended, prognosis: mostly favorable prognosis
	*TP63*-R (FISH)	Prognostically relevant, poor outcome, not required for diagnosis
	*JAK1, JAK3, STAT3* and *MSC* mutations	JAK-STAT pathway alterations, potential therapeutic target
	Rearrangements in *JAK2, TYK2, ROS1, ERK*	*JAK2*-R associated with anaplastic morphology
	*TP53* and *PRDM1* deletions	Associated with poor prognosis
	Truncated *ERBB4*	Potential therapeutic target
TFH lymphoma	TFH markers (IHC): CD279/PD1, ICOS, CXCL13, CD10, BCL6	Diagnostic, required
	Mutations in *RHOA*^G17V^ and *IDH2*^R172^	Support diagnosis, recommended in difficult cases.
	Mutations in *TET2* and *DNMT3A*	Indicate underlying clonal hematopoiesis, important for pathogenesis
	*ICOS::CD28*, *ITK::SYK*, *VAV1* fusions	Support diagnosis. *ITK::SYK* mostly in follicular type
PTCL, NOS	GEP: two subgroups:	Not incorporated into clinical practice
	PTCL-TBX21, low genomic complexity	TBX21 and/or CXCR3 positive (IHC) *TET1, TET3* and *DNMT3A* mutations, favorable outcome. Cytotoxic T-cell lymphomas cluster in this group.
	PTCL-GATA3, high genomic complexity	GATA3 and/or CCR4 positive (IHC) *TP53, PRDM1, STAT3* and *MYC* alterations, poor outcome
*Primary nodal EBV+ T- and NK-cell lymphoma*	Low genomic instability PD-L1 upregulation Downregulation of EBV miRNAs *TET2, PIK3CD, DDX3X* and *STAT3* mutations 14q11.2 loss	New provisional entity; affects mainly elderly and immunocompromised patients. Dismal prognosis

*ALCL*, anaplastic large T-cell lymphoma; *TFH*, Follicular helper T-cell; *PTCL*, *NOS*, peripheral T-cell lymphoma, not otherwise specified; *IHC*, immunohistochemistry; *FISH*, fluorescence in situ hybridization; *R*, rearrangement; *GEP*, gene expression profiling

### Anaplastic large cell lymphoma

Systemic ALCL is divided in two groups based on the presence or absence of *ALK*-R on chromosome 2p23. The most frequent partner gene is *NPM1* on chromosome 5q35.1, followed by a variety of other fusion partners that encode oncogenic ALK fusion proteins. The demonstration of *ALK*-rearrangement or protein by immunohistochemistry is diagnostic of the disease and has allowed the recognition of several histologic variants, including the common variant (60% of the cases), the lymphohistiocytic variant (10%), the small-cell variant (5-10%), the Hodgkin-like variant (3%) and cases with more than one pattern [25]. Constitutive ALK activation promotes tumorigenesis through the activation of multiple signaling pathways such as PLC*γ*, PI3K-AKT1, JAK-STAT, mTOR, and MEK-ERK [15, 78]. Most ALCL have clonally rearranged TR genes despite the failure to express TR molecules on the cell surface [9]. The mutational landscape includes recurrent mutations of *TP53*, epigenetic modifiers, and genes in the TR pathway [53]. More recently, it has been shown that ALK-positive ALCL has recurrent missense mutations in the extracellular domain of *NOTCH1* (12%; distinct from the truncating mutations of the PEST domain in B-cell lymphomas), a potential therapeutic target [48].

**Fig. 5:**
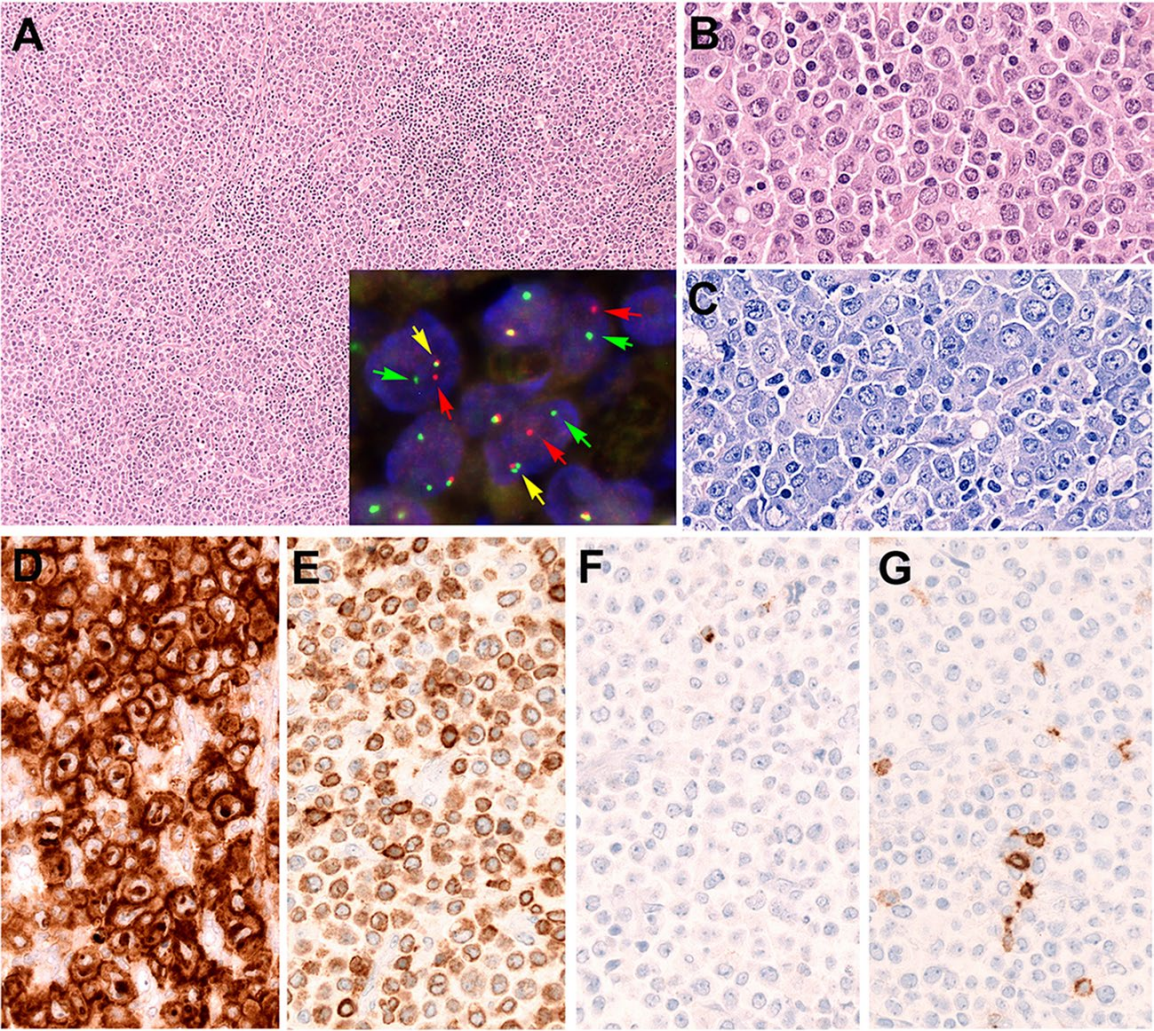
Anaplastic large T-cell lymphoma with *DUSP22* rearrangement. **A** H&E stain shows a lymph node with diffuse infiltration of large anaplastic cells (original magnification 100×). Insert: FISH demonstrates a *DUSP22* break with 1 colocalization signal (yellow arrow) and 1 split signal (green and red arrows) consistent with gene rearrangement. FISH analysis was performed using LSI Dual color break-apart probe from Vysis abbot Molecular. **B** Higher magnification shows the monotonous, anaplastic cytology of the tumor cells. **C** Giemsa stain highlights the blastic chromatin with multiple nucleoli and abundant basophilic cytoplasm (**B**, **C** original magnification 400×). **D**, **E** The tumor cells show a strong characteristic CD30 stain (**D**). The cells are positive for CD3 (**E**) but remain negative for perforin (**F**), TIA1 and granzyme B (not shown), and CD5 (**G**) (original magnification 400×)

ALK-negative ALCL, by definition, lacks *ALK* rearrangement, and although it is considered a single disease entity, molecular data indicates the existence of several distinct subgroups [73]. Similar to ALK+ALCL, activation of the JAK-STAT signaling pathway seems to play an important role and is in many cases secondary to *STAT3*, *JAK1*, and *JAK3* mutations, but also through rearrangements involving *ROS1*, *TYK2* of *FRK*. Two recurrent chromosomal translocations with characteristic clinicopathological features are recognized. ALK-negative ALCL with *DUSP22*-R (20–30%), has monomorphic cytology, absence of cytotoxic molecules and PD-L1 expression, lacks JAK-STAT pathway activation, has a characteristic gene expression signature and recurrent *MSC* mutations [55, 56]. The 2022 ICC considers ALK-negative ALCL with *DUSP22*-R a genetic subtype of ALK-negative ALCL with usually favorable outcome [12, 26]. FISH analysis for *DUSP22* is recommended to identify this genetic subgroup (Fig. 5). The second recurrent translocation involves *TP63* (5–8%) which encodes a p63 fusion protein and is associated with aggressive clinical behavior and poor outcome [73]. Truncated ERBB4 overexpression is observed in 24% of ALK-negative ALCL and represents a potential therapeutic target [89].

### Follicular helper T-cell lymphoma

**Fig. 6:**
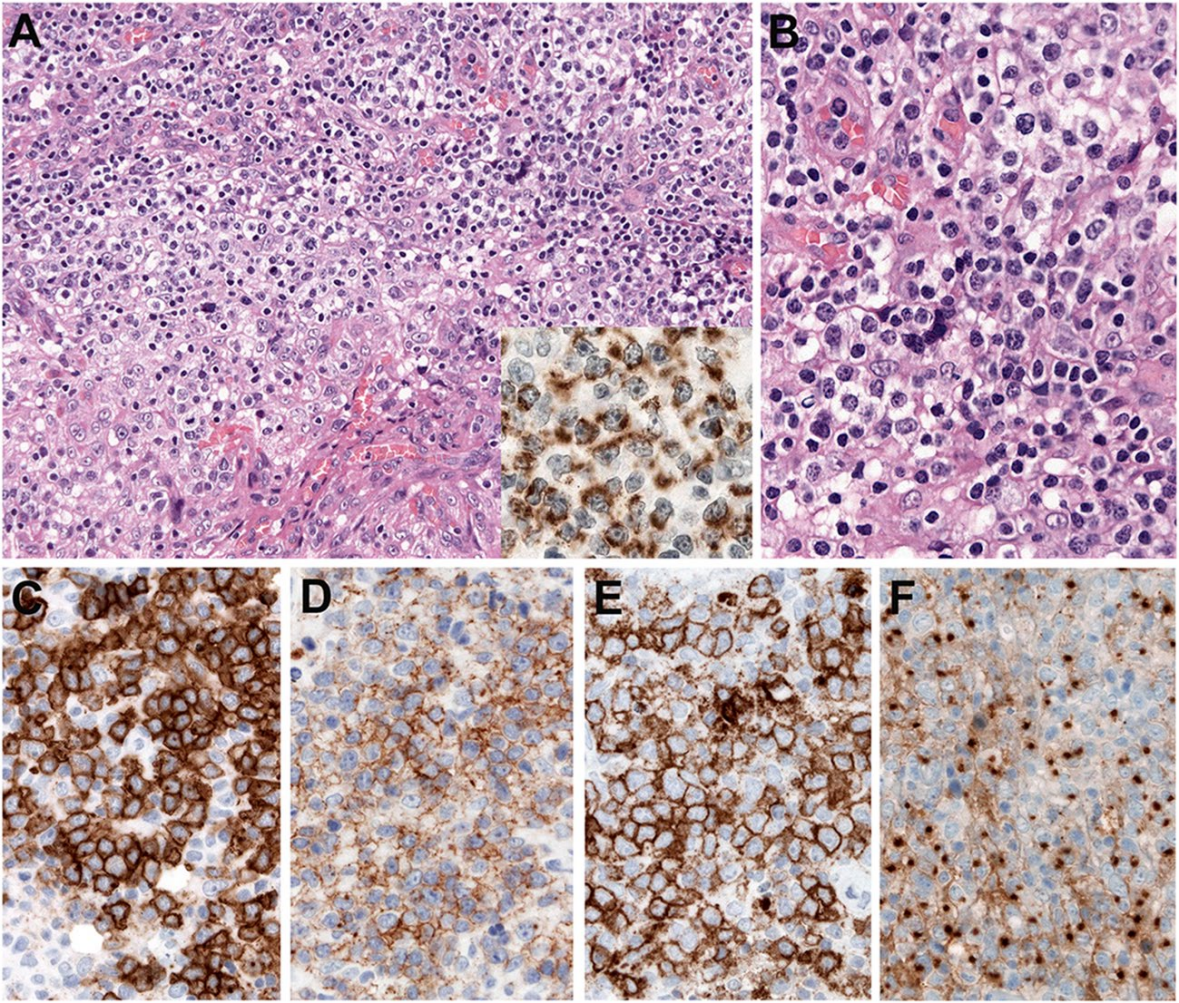
Angioimmunoblastic T-cell lymphoma with *IDH2*^R172*K*^ c.515G>C mutation. **A** H&E stain shows a lymph node with proliferation of large cells with abundant clear cytoplasm. (original magnification, 200x). Insert: the case is stained with the specific antibody against the IDH2^R172K^ mutation. This specific variant is observed in 25–30% of all cases with *IDH2*^R172^ mutation. The paranuclear cytoplasmic positivity is characteristic of the antibody (original magnification, 400×). The variant allelic frequency of this case was 30% by NGS. **B** Higher magnification to show the cytology of the clear cells (original magnification, 400×). **C**–**F** The clear cells are strongly positive for CD10 (**C**), PD1 (**D**), ICOS (**E**), and CXCL13 (**F**). (original magnification, 400×)

TFH lymphoma has distinctive clinical, morphological, immunophenotypic, and genetic features. Angioimmunoblastic T-cell lymphoma (AITL) is the prototype and best-characterized subtype [26]. The mutational landscape of TFH lymphoma includes genes involved in epigenetic pathways such as *TET2* (up to 90%), *IDH2* (20–45%), and *DNMT3A* (20–30%), mutations in the small GTPase *RHOA* (50–70%) and mutations in the TR signaling pathway including *PLCG1*, *CD28*, *FYN*, and *VAV1* [105]. Recurrent translocations have also been identified including *ICOS::CD28*, *ITK::SYK*, and *VAV1* fusions [23]. These genetic alterations are seen across the morphological spectrum of TFH lymphoma supporting the unifying concept of one disease entity. However, *IDH2* mutations are exclusively identified in AITL with characteristic morphology (medium to large cells with clear cytoplasm) and immunophenotype (strong CD10 and CXCL13 expression) (Fig. 6) [97], whereas *ITK::SYK* fusions are mostly identified in the follicular type [98]. *TET2* and *DNMT3A* are the most frequent mutations observed in clonal hematopoiesis (CH) [29], suggesting that CH plays an important role in the pathogenesis of TFH [52]. CH also explains the association of myeloid neoplasms with TFH lymphoma, particularly after cytotoxic therapy [52]. TFH lymphoma is probably the only T-cell lymphoma where mutational analysis is currently recommended to support the diagnosis [12]. In difficult cases, the presence of *RHOA* and/or *IDH2* mutations supports the diagnosis of TFH lymphoma. In the interpretation of molecular findings, it is important to recognize the low allelic frequency often observed in *ROHA* and *IDH2* mutations (VAF <5%) and to avoid misinterpretation of *TET2* and *DNMT3A* as tumor-specific mutations [97].

### Peripheral T-cell lymphoma, not otherwise specified

PTCL, NOS represents a heterogenous group of lymphomas with variable morphology, immunophenotype, cytogenetics and molecular features. The diagnosis of PTCL, NOS often requires the confirmation through TR clonality analysis, supported by mutational analysis [100]. Recently, 2 molecular subgroups were identified through GEP analysis, namely PTCL-TBX21 and PTCL-GATA3 [38]. PTCL-TBX21 is characterized by low genomic complexity, higher frequency of epigenetic modifier genes (*TET1*, *TET3*, *DNMT3A*), and more favorable outcome. In contrast, PTCL-GATA3 is associated with high genomic complexity, gains in *STAT3* and *MYC*, frequent mutations in *TP53* and *PRDM1*, and poor outcome [36]. A novel immunohistochemical algorithm has been proposed to reproduce these two molecular groups using 4 antibodies; TBX21, CXCR3, GATA3, and CCR4 [5]. This algorithm needs validation but likely will be integrated in routine diagnosis in the future. Cytotoxic PTCLs are included within PTCL, NOS and molecularly cluster mainly in the PTCL-TBX21 group.

Primary nodal EBV-positive T- or NK-cell lymphoma is now separated from PTCL, NOS and is considered a provisional entity in the ICC [12, 79]. By definition, it is a systemic disease without nasopharyngeal involvement and affecting mainly elderly or immunocompromised patients. The most common mutated genes are *TET2*, *PIK3CD*, *DDX3X*, and *STAT3*. A characteristic finding is recurrent losses of 14q11.2 where the TRA/D genes are located, supporting the T cell derivation of this lymphoma [69].

### Genetic alterations with potential relevance for the diagnosis of extranodal and leukemic/disseminated T- and NK-cell lymphomas

Some neoplasms derived from mature T and NK cells, mostly from innate immune cells, affect predominantly extranodal sites [21]. These include extranodal NK/T-cell lymphoma (ENKTCL), nasal type, the group of gastrointestinal lymphomas, hepatosplenic T-cell lymphomas (HSTCL), and the rare group of γδ T-cell lymphomas and subcutaneous panniculitis-like T-cell lymphomas (SPTCL). Other neoplasms involve predominantly the peripheral blood and bone marrow such as T-cell large granular lymphocytic leukemia (T-LGLL), T-cell prolymphocytic leukemia (T-PLL), adult T-cell lymphoma/leukemia (ATLL) and aggressive NK-cell leukemia (ANKL). The diagnosis of these diseases is based on their characteristic clinicopathologic features. Besides TR clonality assessment (with the exception of ENKTCL if derived from NK-cells and ANKL, which lack clonal TR rearrangements), molecular analysis is not required for the diagnosis. Nevertheless, extranodal T- and NK-cell lymphomas share the activation of the JAK/STAT pathway, a potential therapeutic target [20]. In difficult differential diagnosis, the mutational pattern, although not specific, might favor one disease over another (Table 4).

**Table 4:** Genetic findings and biomarkers with potential relevance in extranodal and leukemic/disseminated T- and NK-cell lymphomas

Disease entity	Genetic alterations and biomarkers	Clinical relevance
Enteropathy associated T-cell lymphoma (EATL)	*JAK1, STAT3, TNFAIP3, KMT2D, TET2* mutations	Mutational pattern supports diagnosis over MEITL These genetic alterations are shared with RCD-II, considered a precursor lesion to EATL
Monomorphic epitheliotropic intestinal T-cell lymphoma (MEITL)	*SETD2* alterations, *STAT5B*, *JAK3*, *GNA12*, *TP53* and *MYC* mutations	*SETD2* alterations co-occurred often with *STAT5B* and *JAK3*. Mutational pattern supports diagnosis over EATL
	H3K36me3 (IHC)	Absence of expression good surrogate marker of *SETD2* inactivation
Hepatosplenic T-cell lymphoma	Iso 7q, trisomy 8, *STAT5B*, *STAT3* (rare), *SETD2*, *INO80, TET3, SMARCA2* mutations	*SETD2* alterations co-occurred with mutations in other chromatin modifier genes. Mutational pattern supports diagnosis over T-LGLL
T-cell large granular lymphocytic leukemia (T-LGLL)	*STAT3* and *STAT5B* (rare) mutations.	*STAT5B* mutations mainly associated with indolent CD4+ T-LGLL and a subgroup of CD8+ aggressive T-LGLL
	*STAT3* ^Y640F^	Associated with lower ANC values
	*STAT3* ^N647I^	Associated with lower HGB values
	*STAT3* mutations co-occur with *KMT2D* and *SETD1B*	Important for pathogenesis
T-cell prolymphocytic leukemia (T-PLL)	*TCL1* alterations (FISH) inv(14)(q11q32); t(14;14)(q11;q32) MCTP1 (X;14)(q28;q11)	Diagnostic, in most cases not required. TCL1 (IHC) diagnostic, required
	*STAT5B, JAK1*, and *JAK3* mutations	JAK-STAT pathway activation, important for pathogenesis
Subcutaneous panniculitis-like T-cell lymphoma	*HAVCR2* germline mutation	Younger patients (<30 years), often associated with HLH and worse prognosis
	*HAVCR2* ^Y82C^	East Asia, Polynesian ancestry
	*HAVCR2* ^I97M^	European population
Extranodal NK/T-cell lymphoma, nasal type	Del(6q), *STAT3*, *TP53, DDX3X, BCOR, TET2* mutations	JAK-STAT pathway activation, important for pathogenesis.
	EBER1 ISH	Diagnostic, required
Aggressive NK-cell leukemia	Del(6q), del(11q), *STAT3*, *TP53, DDX3X, BCOR, TET2* mutations	JAK-STAT pathway activation, important for pathogenesis.
	EBER ISH	Diagnostic, required

*RCD-II*, refractory celiac disease type II; FISH: fluorescence in situ hybridization; *IHC*, immunohistochemistry; *ANC*, absolute neutrophils count; *HGB*, hemoglobin; *HLH*, hemophagocytic lymphohistiocytosis; *EBER*, Epstein-Barr virus encoded small RNA; *ISH*, in situ hybridization

Enteropathy-associated T-cell lymphoma (EATL) is characterized by frequent mutations in the JAK/STAT pathway, most commonly involving *JAK1* and *STAT3* and rarely *JAK3*, *STAT5B*, *TYK2*, or *SOCS1*. *KMT2D* and *TET2* are also frequently mutated in EATL [17]. In contrast, monomorphic epitheliotropic intestinal T-cell lymphoma (MEITL) is characterized by mutations or deletions of *SETD2* (>90%), resulting in reduced or absent of expression of lysine 36 in histone H3 (H3K36me3). Other characteristic co-occurrent mutations are *STAT5B* (<60%) and *JAK3* (35–50%). The absence of H3K36me3 expression by immunohistochemistry is an excellent surrogate marker for *SETD2* inactivation [81].

ENKTCL and ANKL share a similar mutational profile, frequently affecting the JAK/STAT pathway (*STAT3*, *STAT5B*, *JAK3*), epigenetic regulators (*BCOR*, *KMT3D*, *ARID1A*, *EP300*), tumor suppressor genes (*TP53*, *MGA*) and the RNA helicase *DDX3X* [21, 61, 79].

The differential diagnosis of HSTCL, T-LGLL, and γδ T-cell lymphoproliferations is not always straightforward. Characteristic genetic alterations of HSTCL are the presence of isochromosome 7q (80%) and trisomy 8 (50%) regardless of the αβ/γδ derivation [77]. Inactivating mutations in *SETD2* (70%), and in other chromatin-modifying genes (*INO80*, *TET3*, *SMARCA2*) are frequently identified (62%). Other mutations frequently observed are *STAT5B* (31%), *STAT3* and *PIK3CD* and rare cases also carry *TP53*, *URB5*, and *IDH2* (5–10%) mutations [20]. In contrast, T-LGLL carry *STAT3* mutations in up to 50% of the cases and co-occur frequently with *KMT2D* and *SETD1B* mutations [14]. *STAT5B* mutations are mainly associated with the indolent CD4+ T-LGLL and a subgroup of CD8+ aggressive T-LGLL [8].

T-PLL is characterized by 14q chromosomal inversions with breakpoints in the long arm at q11 and q32 seen in 80% of the cases. These alterations involve the TCL1 family genes and less often *MTCP1* gene, which is homologous to *TCL1A/B* at Xq28. Recurrent mutations in the JAK/STAT pathway are observed up to 75% of the cases. Monoallelic deletions and/or mutations of *ATM* are common [20, 99]. Demonstration of *TCL1* chromosomal alteration by FISH or TCL1 expression in T-cells by immunohistochemistry is diagnostic of T-PLL.

Germline mutations of the *HAVCR2* gene have been demonstrated in patients with SPTCL [28]. Two hot spot mutations have been identified, one predominates in East Asia (*HAVCR2*^Y82C^) and the other in Europeans (*HAVCR2*^I97M^). *HAVCR2* encodes for TIM3, an inhibitory receptor expressed on interferon-γ producing T-cells involved in regulating inflammation. Patients with *HAVCR2* mutation are younger (<30 years), often associated with hemophagocytic lymphohistiocytosis (HLH) and worse prognosis [95]. The investigation of *HAVCR2* mutations may allow risk stratification and provide a target for therapy.

## Conclusions and outlook

As evidenced by the increasing emphasis on molecular features in contemporary lymphoma classifications, molecular diagnostics is gaining in importance for everyday decision-making both for the pathologist and the treating clinician [2, 12, 20]. Although many lymphomas still can be classified reliably with the morphology and immunophenotyping and in part investigation of single markers, we will see an increasing use of multigene panels or whole exome sequencing for detailed genetic profiling and probably a replacement of different techniques such as PCR and FISH by a comprehensive NGS-based approach. Newer techniques including whole genome and single-cell sequencing and methylation and chromatin profiling likely will also enter clinical practice for specific applications [21]. Detection of cell-free tumor DNA in the peripheral blood, so-called liquid biopsy shows promise for the follow-up of patients with aggressive lymphomas under treatment, although sensitivity and standardization issues and potential pitfalls such as clonal evolution and clonal hematopoiesis require more work before broad clinical application [83].
